# Association Between Geriatric Assessment Scores and Corneal Biomechanical Parameters in Patients with Glaucoma

**DOI:** 10.3390/biomedicines14071546

**Published:** 2026-07-10

**Authors:** Yuto Yoshida, Yuri Fujino, Yuya Kato, Mayumi Furue, Hinako Ohtani, Chisako Ida, Kana Murakami, Mizuki Koike, Keigo Takagi, Kazunobu Sugihara, Masaki Tanito

**Affiliations:** Department of Ophthalmology, Shimane University Faculty of Medicine, Izumo 693-8501, Japan; y-yoshida@juntendo.ac.jp (Y.Y.);

**Keywords:** geriatric assessments, corneal biomechanical parameters, corneal hysteresis, corneal resistance factor, Geriatric 8, mini-Cog, Charlson Comorbidity Index, frailty, glaucoma

## Abstract

**Background/Objectives**: Multiple age-related systemic conditions, including frailty, cognitive impairment, and comorbid diseases, have been suggested to be associated with glaucoma. However, their relationship with corneal biomechanical properties in patients with glaucoma remains unclear. **Methods**: This retrospective cross-sectional study included patients with glaucoma who attended the Department of Ophthalmology at Shimane University Hospital between May 2019 and August 2024. Corneal biomechanical parameters, including corneal hysteresis (CH), corneal resistance factor (CRF), corneal-compensated intraocular pressure (IOPcc), and Goldmann-correlated intraocular pressure (IOPg), were measured using the Ocular Response Analyzer (ORA; Reichert Technologies, Depew, NY, USA). Geriatric assessments, including the Geriatric 8 (G8), Mini-Cog, and Charlson Comorbidity Index (CCI), were also evaluated. Associations between geriatric assessment measures and ocular parameters were examined using multivariable linear mixed-effects models adjusted for age, sex, medication score, and glaucoma subtype. **Results**: A total of 280 patients (456 eyes) were included. The mean age was 70.2 ± 11.1 years, and 126 patients (45.0%) were women. In multivariable linear mixed-effects models, lower G8 scores were significantly associated with lower CRF (β = 0.18, 95% CI: 0.05 to 0.30), lower IOPcc (β = 0.55, 95% CI: 0.18 to 0.92), and lower IOPg (β = 0.62, 95% CI: 0.25 to 1.00). In contrast, no significant association was observed between G8 scores and CH (β = −0.02, 95% CI: −0.12 to 0.09). Neither Mini-Cog nor CCI was significantly associated with any ocular parameters. **Conclusions**: In patients with glaucoma, frailty may be associated with corneal biomechanical properties, particularly CRF and intraocular pressure-related parameters.

## 1. Introduction

With advancing population aging, the burden of frailty, cognitive impairment, and chronic comorbidities continues to rise [1,2,3]. Emerging evidence suggests that biological aging does not occur within a single organ alone; rather, aging in one organ system may affect the decline of others, reflecting an interconnected multiorgan aging network [4,5]. In this context, glaucoma should be considered not only an ocular disease, but also part of the broader systemic health challenges faced by older adults [6,7].

Several studies have reported that frailty is significantly associated with an increased risk of glaucoma and is also correlated with the severity of visual field impairment [8,9]. A previous meta-analysis highlighted a significant relationship between glaucoma and a higher risk of dementia, including all-cause dementia, Alzheimer’s disease, vascular dementia, and mild cognitive impairment (MCI) [10]. Additionally, glaucoma is closely linked to chronic systemic comorbidities, such as cardiovascular, metabolic, and neurodegenerative diseases [11,12,13].

However, evidence regarding the relationship between ocular biomarkers and age-related systemic conditions remains limited [14,15,16,17,18,19,20]. Corneal biomechanical parameters, such as corneal hysteresis (CH) and corneal resistance factor (CRF), may reflect age-related alterations in collagen structure and tissue viscoelasticity [21,22,23]; however, their associations with frailty, cognitive function, and comorbidity burden remain largely unexplored. Therefore, we investigated the associations between geriatric assessment measures and corneal biomechanical parameters in patients with glaucoma.

## 2. Materials and Methods

### 2.1. Participants and Study Design

This cross-sectional study included patients with glaucoma who attended the ophthalmology department at Shimane University Hospital between May 2019 and August 2024. Inclusion criteria were as follows: assessment with the Ocular Response Analyzer (ORA; Reichert Technologies, Depew, NY, USA) and geriatric evaluation, including Geriatric 8 (G8), Mini-Cog, and Charlson Comorbidity Index (CCI), performed on the same day. Measurements with a waveform score < 7 were excluded from the analysis because of insufficient measurement reliability.

This study was performed in accordance with the principles of the Declaration of Helsinki and received approval from the Institutional Review Board of Shimane University Hospital (IRB No. 20200228-2; approval date of updated protocol: 27 April 2026). The IRB waived the requirement for written informed consent. Instead, study information was disclosed on the participating institution’s website, and participants were provided with the opportunity to decline participation through an opt-out process.

### 2.2. Assessment with the Ocular Response Analyzer (ORA)

The Ocular Response Analyzer (ORA) was used to obtain the following corneal biomechanical parameters: CH, CRF, corneal-compensated intraocular pressure (IOPcc), and Goldmann-correlated intraocular pressure (IOPg). This device evaluates IOP and corneal biomechanical properties by analyzing the inward and outward corneal deformation responses induced by an air pulse over approximately 20 ms [24,25]. Detailed definitions and calculation methods for each parameter have been described in our previous methodological report [26]. Three measurements were obtained for each eye, and the dataset with the highest waveform score was selected for analysis.

### 2.3. Geriatric Assessments (GAs)

Geriatric assessments (GAs) are used across multiple clinical specialties to evaluate the overall health status of older adults and support individualized management strategies. In our institution, patients routinely underwent GAs including G8, Mini-Cog, and CCI. The G8 was used as a screening measure of frailty and vulnerability in older adults. This 8-item instrument assesses reduced food intake and recent weight loss over the preceding 3 months, impaired mobility, cognitive or psychological problems, body mass index, polypharmacy, self-rated health, and age. Total scores range from 0 to 17, with lower scores indicating greater vulnerability; a score of ≤14 is commonly considered suggestive of frailty [14,15,17]. The Mini-Cog testing is a brief screening instrument for cognitive function that combines a three-item recall task (0–3 points) and a scored clock-drawing component (0–2 points). Total scores of 2 or less are commonly used as a threshold indicating possible cognitive impairment or dementia [16]. The CCI is a validated tool used to assess overall comorbidity burden by assigning weighted scores to multiple chronic diseases, including cardiovascular, pulmonary, hepatic, renal, metabolic, and malignant conditions, as well as dementia [18]. These instruments assess complementary domains of geriatric health, including frailty, cognitive function, and comorbidity burden. Together, they provide a multidimensional assessment of older adults.

### 2.4. Statistical Analysis

Continuous variables are presented as the mean ± standard deviation (SD), whereas categorical variables are summarized as number (%). Correlations among age, GA measures (G8, Mini-Cog, and CCI), and ocular parameters (CH, CRF, IOPg, and IOPcc) were evaluated using Spearman’s rank correlation coefficients and are presented in a correlation matrix.

Associations between geriatric assessment measures and ocular parameters were examined using multivariable linear mixed-effects models with a random intercept for each participant to account for within-subject correlation. These analyses were performed separately for each ocular parameter, including CH, CRF, IOPcc, and IOPg. All models were adjusted for age, sex, G8, CCI, Mini-Cog score, medication score, and glaucoma subtype. Glaucoma subtype was categorized as primary open-angle glaucoma (POAG), exfoliation glaucoma (EXG), or others. Additional stratified analyses were performed separately in the POAG and EXG subgroups. Glaucoma subtype was determined by a glaucoma specialist (M.T.) on the same day as the GA and ORA examination. The diagnosis was based on a comprehensive ophthalmic evaluation, including slit-lamp biomicroscopy, gonioscopy, stereoscopic optic disc assessment, optical coherence tomography, and standard automated perimetry. The medication score was calculated by assigning one point for each active ingredient in topical glaucoma medications and one point for each oral acetazolamide tablet.

As sensitivity analyses, we performed two additional multivariable linear mixed-effects models. First, mean deviation (MD), an indicator of glaucoma severity, was additionally included as a continuous covariate to assess the potential influence of glaucoma severity. Second, age was excluded from the models because it is a component of the G8 score, in order to evaluate the potential influence of multicollinearity and overadjustment.

Two-sided *p* values < 0.05 were considered statistically significant. All statistical analyses were performed using R software (version 4.5.1; R Foundation for Statistical Computing, Vienna, Austria).

## 3. Results

A total of 280 patients (456 eyes) with glaucoma were included in this study. As shown in Table 1, the mean age of the participants was 70.2 ± 11.1 years, and 126 participants (45.0%) were women. The G8, CCI, and Mini-Cog scores were 14.3 ± 1.79, 0.31 ± 0.76, and 3.61 ± 1.85, respectively. At the eye level, IOPcc, IOPg, CH, and CRF were 17.0 ± 5.80, 15.1 ± 5.85, 9.17 ± 1.48, and 9.25 ± 1.76 mmHg, respectively. Regarding glaucoma subtype, 253 eyes (55.5%) had POAG, 73 eyes (16.0%) had EXG, and 130 eyes (28.5%) were classified as others.

**Figure 1:**
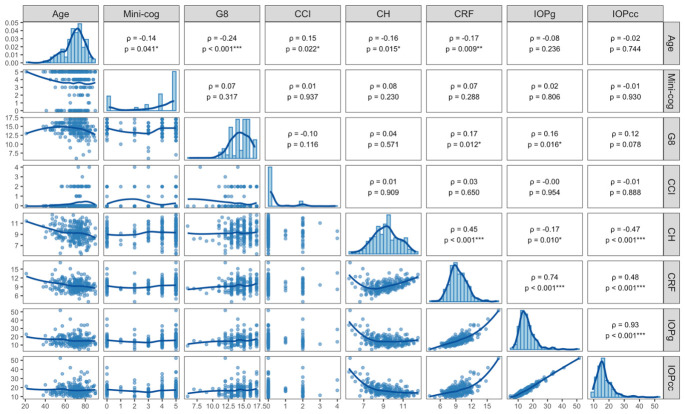
Correlations among geriatric assessment measures, corneal parameters, and intraocular pressure. Scatter plots are shown for each pair of variables, and histograms on the diagonal indicate the distribution of each variable. Spearman correlation coefficients (ρ) and corresponding *p* values are presented. * *p* < 0.05, ** *p* < 0.01, *** *p* < 0.001. CCI, Charlson Comorbidity Index; CH, corneal hysteresis; CRF, corneal resistance factor; IOPg, Goldmann-correlated intraocular pressure; IOPcc, corneal-compensated intraocular pressure.

**Figure 2:**
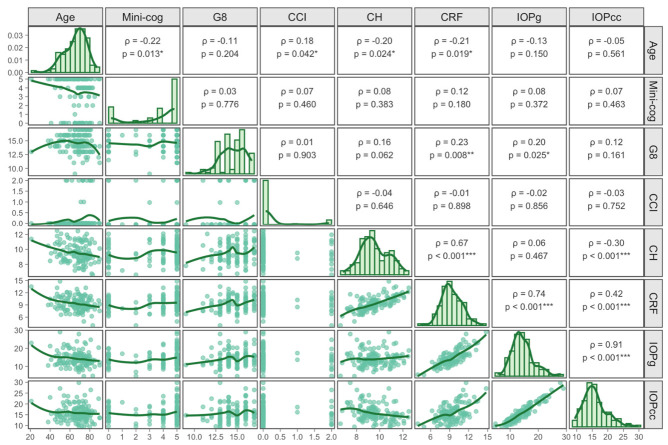
Correlations among geriatric assessment measures, corneal parameters, and intraocular pressure in primary open-angle glaucoma. Scatter plots are shown for each pair of variables, and histograms on the diagonal indicate the distribution of each variable. Spearman correlation coefficients (ρ) and corresponding *p* values are presented. * *p* < 0.05, ** *p* < 0.01, *** *p* < 0.001. CCI, Charlson Comorbidity Index; CH, corneal hysteresis; CRF, corneal resistance factor; IOPg, Goldmann-correlated intraocular pressure; IOPcc, corneal-compensated intraocular pressure.

**Figure 3:**
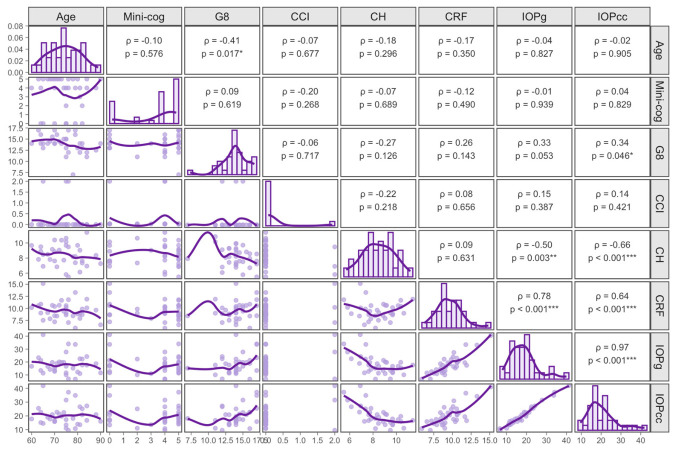
Correlations among geriatric assessment measures, corneal parameters, and intraocular pressure in exfoliation glaucoma. Scatter plots are shown for each pair of variables, and histograms on the diagonal indicate the distribution of each variable. Spearman correlation coefficients (ρ) and corresponding *p* values are presented. * *p* < 0.05, ** *p* < 0.01, *** *p* < 0.001. CCI, Charlson Comorbidity Index; CH, corneal hysteresis; CRF, corneal resistance factor; IOPg, Goldmann-correlated intraocular pressure; IOPcc, corneal-compensated intraocular pressure.

Figure 1 shows the correlations among age, GA measures, corneal parameters, and IOP. Age was significantly correlated with the following variables: Mini-Cog (ρ = −0.14, *p* = 0.041), G8 (ρ = −0.24, *p* < 0.001), CCI (ρ = 0.15, *p* = 0.022), CH (ρ = −0.16, *p* = 0.015), and CRF (ρ = −0.17, *p* = 0.009). G8 was significantly correlated with CRF (ρ = 0.17, *p* = 0.012) and IOPg (ρ = 0.16, *p* = 0.016). Among the ORA-derived parameters, CH, CRF, IOPcc, and IOPg were all significantly correlated with one another. In the POAG subgroup, the correlation analysis showed patterns broadly consistent with those observed in the overall cohort (Figure 2). In the EXG subgroup, correlations among the ORA-derived parameters showed a similar pattern, whereas G8 was significantly correlated only with age (ρ = −0.41, *p* = 0.017) and IOPcc (ρ = 0.34, *p* = 0.046) (Figure 3).

Table 2 shows the associations between GA measures and ocular parameters using multivariable linear mixed-effects models. Older age was significantly associated with lower CH (β = −0.03, 95% confidence interval [CI]: −0.05 to −0.01) and lower CRF (β = −0.03, 95% CI: −0.05 to −0.01). Lower G8 scores were significantly associated with lower CRF (β = 0.18, 95% CI: 0.05 to 0.30), lower IOPcc (β = 0.55, 95% CI: 0.18 to 0.92), and lower IOPg (β = 0.62, 95% CI: 0.25 to 1.00). Higher medication scores were significantly associated with lower CH (β = −0.17, 95% CI: −0.25 to −0.09) and higher IOPcc (β = 0.33, 95% CI: 0.00 to 0.66). Compared with POAG, EXG was significantly associated with lower CH (β = −0.69, 95% CI: −1.07 to −0.30), higher CRF (β = 0.57, 95% CI: 0.11 to 1.02), higher IOPcc (β = 4.11, 95% CI: 2.60 to 5.61), and higher IOPg (β = 3.94, 95% CI: 2.39 to 5.48).

Table 3 presents the subgroup analysis in patients with POAG. Older age was significantly associated with lower CH (β = −0.03, 95% CI: −0.05 to −0.01) and lower CRF (β = −0.03, 95% CI: −0.05 to −0.01). Lower G8 scores were significantly associated with lower CRF (β = 0.25, 95% CI: 0.07 to 0.42) and lower IOPg (β = 0.60, 95% CI: 0.10 to 1.09). Higher medication scores were significantly associated with lower CH (β = −0.19, 95% CI: −0.28 to −0.09) and higher IOPcc (β = 0.41, 95% CI: 0.03 to 0.78). In the EXG subgroup, no significant associations were identified between GA measures and corneal biomechanical parameters (Table 4).

Additional adjustment for MD, an indicator of glaucoma severity, did not alter the associations between geriatric assessment scores and corneal biomechanical parameters (Table 5). G8 score remained significantly associated with CRF (β = 0.18, 95% CI: 0.05 to 0.31), IOPg (β = 0.61, 95% CI: 0.21 to 1.02), and IOPcc (β = 0.53, 95% CI: 0.14 to 0.93), whereas no significant association was observed with CH. In addition, worse MD was independently associated with lower CH and CRF, whereas no significant associations were observed with IOPg or IOPcc.

In a sensitivity analysis excluding age from the multivariable models, the associations between geriatric assessment scores and corneal biomechanical parameters remained largely unchanged compared with the primary analysis (Table 6), indicating that the main findings were robust to the exclusion of age.

## 4. Discussion

This cross-sectional study investigated the associations between GAs and corneal biomechanical parameters. G8 scores were significantly associated with CRF, IOPcc, and IOPg, whereas no significant association was observed with CH. In contrast, Mini-Cog and CCI were not significantly associated with any ocular parameters measured by the ORA. These findings suggest that systemic frailty may be reflected in ocular biomechanical characteristics.

Older adults with glaucoma were significantly more likely to exhibit frailty [8,27]. Mendelian randomization analyses suggested that genetically predicted frailty was associated with an increased risk of several glaucoma subtypes, including POAG and EXG [8]. A previous study reported that frailty in glaucoma patients may increase the risk of progressive visual field loss [28]. In contrast, studies examining the relationship between frailty and ophthalmic biomarkers in glaucoma remain limited. Our group previously demonstrated that cognitive frailty was significantly associated with OCT-derived retinal biomarkers, including inner macular thickness, in patients with glaucoma [29]. In the present study, we investigated the association between frailty and corneal biomechanical parameters in glaucoma patients, which to our knowledge has not been previously reported. Although frailty has been widely recognized to be associated with age [30], the significant association between greater frailty and lower CRF remained even after adjustment for age in our analysis. Given these findings, systemic frailty may be reflected in ocular biomarkers. Further studies are needed to clarify the impact of frailty on ophthalmic biomarkers in patients with glaucoma.

One possible explanation for our findings is that frailty may influence corneal biomechanical properties through biological aging-related alterations. Biological aging is accompanied by extracellular matrix remodeling and age-related changes in collagen organization, leading to alterations in tissue mechanical properties [31]. Similarly, the cornea may undergo age-related structural and compositional changes, including collagen glycation, non-enzymatic collagen crosslinking, and keratocyte loss, which may contribute to alterations in corneal biomechanical properties [32]. Therefore, systemic frailty may contribute to alterations in corneal biomechanical properties through age-related remodeling of the corneal extracellular matrix and collagen architecture. Additionally, oxidative stress may represent one potential mechanism underlying these age-related extracellular matrix alterations. Previous studies have reported an association between frailty and increased oxidative stress [33,34]. Furthermore, systemic antioxidant capacity has been shown to be positively associated with corneal biomechanical parameters, including CH and CRF [22], suggesting that oxidative stress may contribute to alterations in corneal biomechanics. Experimental studies have also shown that oxidative stress may accelerate keratocyte apoptosis, suppress collagen expression, and promote extracellular matrix degradation through modulation of matrix metalloproteinase-2 (MMP-2) and tissue inhibitor of metalloproteinase-1 (TIMP-1) [21,23]. Together, these findings suggest that oxidative stress may contribute to age-related remodeling of the corneal extracellular matrix, although the underlying mechanisms remain to be fully elucidated. In the present study, frailty was significantly associated with CRF but not with CH; the reasons for this discrepancy remain unclear. Further studies are warranted to elucidate the underlying mechanisms of this differential association.

The present study suggests that corneal biomechanical properties may differ between EXG and POAG. Previous studies have reported that CH is lower in EXG than in POAG [35,36]. Consistent with these findings, CH was significantly lower in EXG compared with POAG in the present study (Table 2). A previous study using CorVis ST demonstrated that eyes with EXG exhibit higher stress–strain index (SSI) values compared with POAG (1.42 ± 0.27 vs. 1.29 ± 0.26; *p* = 0.022), indicating increased structural stiffness of the cornea. In addition, the speed of corneal movement during the second applanation has been reported to be lower in EXG, suggesting altered dynamic recovery behavior [37]. Taken together, these findings suggest that EXG may be characterized by increased corneal stiffness and altered viscoelastic behavior, which could contribute to the lower CH observed in EXG compared with POAG. In contrast, CRF was higher in EXG compared with POAG in the present study. This finding may be partly explained by the higher IOP observed in EXG. As shown in Table 2, IOP was significantly higher in EXG than in POAG, which likely led to an increase in the first applanation pressure (P1). Given that CRF is calculated as a weighted function of P1 and the second applanation pressure (P2) [26], with a greater contribution from P1, the elevated P1 in EXG may have contributed to the higher CRF values. Because pseudoexfoliation syndrome is increasingly recognized as a systemic extracellular matrix disorder, these distinctive corneal biomechanical features may reflect not only local ocular changes but also systemic extracellular matrix abnormalities. Further studies are warranted to clarify whether these systemic alterations contribute to corneal biomechanical changes in EXG.

Frailty reflects a state of systemic physiological decline; however, its association with IOP has not been well investigated. The present study suggests that lower levels of frailty are associated with higher IOP. As shown in Table 2, lower G8 scores were significantly associated with lower IOPcc (β = 0.55, 95% CI: 0.18 to 0.92) and lower IOPg (β = 0.62, 95% CI: 0.25 to 1.00). Frailty is closely related to aging, and several population-based studies in Japan have reported an inverse association between age and IOP, with older individuals showing lower IOP [38,39,40]. The present findings are consistent with this pattern. It is possible that frailty influences IOP through alterations in aqueous humor dynamics. Additionally, age-related alterations in corneal biomechanical properties, including CH and CRF, may also contribute to changes in measured IOP. Further studies are required to clarify this relationship between frailty and IOP.

This study has several limitations. First, causal relationships cannot be established given the cross-sectional design of this study. Future cohort studies are warranted to further clarify the association between frailty and corneal biomechanical properties. Second, residual confounding may be present. Several systemic factors that may influence both frailty and corneal biomechanics, including body mass index, nutritional status, systemic inflammatory conditions, steroid exposure, and systemic medication use, were not available in the present study. In addition, GAs may be influenced by socioeconomic status, including factors such as education, income, and access to healthcare [1,41,42,43,44]. Future studies should account for these factors to more accurately elucidate the association. Third, the generalizability of the findings may be limited, as this was a single-center study conducted at a university hospital. The study population may have included a higher proportion of patients with more severe glaucoma and poorer geriatric assessment results. Fourth, there may have been potential measurement bias associated with the ORA. However, measurements with a waveform score < 7 were excluded to ensure adequate reliability. Fifth, the clock drawing component of the Mini-Cog may have been influenced by visual impairment. Future studies should consider the use of cognitive screening tools that are less dependent on visual function, such as the Mini-Mental State Examination for visually impaired individuals (MMSE-blind) [45] or the Montreal Cognitive Assessment for visually impaired individuals (MoCA-VI) [46].

## 5. Conclusions

This study demonstrated significant associations between G8 scores and ORA-derived parameters, including CRF, IOPcc, and IOPg. Frailty may be associated with corneal biomechanical properties.

## Figures and Tables

**Table 1:** Baseline characteristics of participants at the individual and eye levels.

Characteristics	
**Individual-level**	**N** **= 280**
Age, year	70.2 ± 11.1
Women, n (%)	126 (45.0%)
Hypertension, n (%)	137 (59.3%)
Diabetes mellitus, n (%)	41 (17.7%)
Systolic blood pressure, mmHg	143.8 ± 28.1
Diastolic blood pressure, mmHg	81.6 ± 17.1
Current smoking, n (%)	27 (9.7%)
G8, points	14.3 ± 1.79
Charlson Comorbidity Index, points	0.31 ± 0.76
Mini-cog, points	3.61 ± 1.85
**Eye-level**	**N = 456 (eyes)**
Right eyes, n (%)	227 (49.8%)
IOPcc, mmHg	17.0 ± 5.80
IOPg, mmHg	15.1 ± 5.85
Corneal hysteresis, mmHg	9.17 ± 1.48
Corneal resistance factor, mmHg	9.25 ± 1.76
Glaucoma subtypes, n (%)	
Primary open-angle glaucoma	253 (55.5%)
Exfoliation glaucoma	73 (16.0%)
Others	130 (28.5%)
Medication scores, points	2.81 ± 1.62
Mean deviation, dB	−6.28 ± 6.73
Axial length, mm	25.3 ± 1.85

IOPg, Goldmann-correlated intraocular pressure; IOPcc, corneal-compensated intraocular pressure.

**Table 2:** Association between geriatric assessment and ocular parameters using multivariable linear mixed-effects models.

Variables	CH	CRF	IOPcc	IOPg
	β (95% CI)	β (95% CI)	β (95% CI)	β (95% CI)
Age (per year)	−0.03 *** (−0.05, −0.01)	−0.03 ** (−0.05, −0.01)	0.02 (−0.04, 0.08)	−0.01 (−0.07, 0.05)
Male (vs. Female)	−0.19 (−0.55, 0.16)	−0.19 (−0.63, 0.25)	0.16 (−1.09, 1.41)	−0.06 (−1.37, 1.25)
G8	−0.02 (−0.12, 0.09)	0.18 ** (0.05, 0.30)	0.55 ** (0.18, 0.92)	0.62 ** (0.25, 1.00)
CCI	0.06 (−0.20, 0.31)	0.08 (−0.24, 0.40)	0.08 (−0.83, 0.98)	0.15 (−0.80, 1.10)
Mini-cog	0.02 (−0.08, 0.11)	0.02 (−0.10, 0.14)	−0.02 (−0.35, 0.31)	0.01 (−0.34, 0.36)
Medication score	−0.17 (−0.25, −0.09)	−0.03 (−0.13, 0.06)	0.33 * (0.00, 0.66)	0.22 (−0.11, 0.56)
EXG (vs. POAG)	−0.69 *** (−1.07, −0.30)	0.57 * (0.11, 1.02)	4.11 *** (2.60, 5.61)	3.94 *** (2.39, 5.48)
Others (vs. POAG)	−0.14 (−0.64, 0.36)	0.00 (−0.61, 0.61)	0.84 (−0.98, 2.67)	0.73 (−1.17, 2.63)

Values are regression coefficients (β) with 95% confidence intervals. All models were adjusted for age, sex, medication score, and glaucoma subtype. * *p* < 0.05, ** *p* < 0.01, *** *p* < 0.001. CI, confidence interval; CCI, Charlson Comorbidity Index; CH, corneal hysteresis; CRF, corneal resistance factor; IOPg, Goldmann-correlated intraocular pressure; IOPcc, corneal-compensated intraocular pressure; EXG, exfoliation glaucoma; POAG, primary open-angle glaucoma.

**Table 3:** Association between geriatric assessment and ocular parameters using multivariable linear mixed-effects models in patients with Primary Open-Angle Glaucoma.

Variables	CH	CRF	IOPcc	IOPg
	β (95% CI)	β (95% CI)	β (95% CI)	β (95% CI)
Age (per year)	−0.03 ** (−0.05, −0.01)	−0.03 ** (−0.05, −0.01)	0.01 (−0.06, 0.07)	−0.03 (−0.09, 0.04)
Male (vs. Female)	−0.19 (−0.62, 0.25)	−0.12 (−0.66, 0.42)	0.32 (−1.14, 1.79)	0.15 (−1.38, 1.69)
G8	0.08 (−0.06, 0.22)	0.25 ** (0.07, 0.42)	0.43 (−0.05, 0.90)	0.60 * (0.10, 1.09)
CCI	0.20 (−0.15, 0.56)	−0.03 (−0.48, 0.41)	−0.76 (−1.96, 0.44)	−0.65 (−1.91, 0.61)
Mini-cog	0.04 (−0.07, 0.16)	0.07 (−0.08, 0.21)	0.03 (−0.36, 0.41)	0.09 (−0.32, 0.49)
Medication score	−0.19 *** (−0.28, −0.09)	−0.07 (−0.19, 0.04)	0.41 * (0.03, 0.78)	0.24 (−0.15, 0.23)

Values are regression coefficients (β) with 95% confidence intervals. All models were adjusted for age, sex, medication score, and glaucoma subtype. * *p* < 0.05, ** *p* < 0.01, *** *p* < 0.001. CI, confidence interval; CCI, Charlson Comorbidity Index; CH, corneal hysteresis; CRF, corneal resistance factor; IOPg, Goldmann-correlated intraocular pressure; IOPcc, corneal-compensated intraocular pressure.

**Table 4:** Association between geriatric assessment and ocular parameters using multivariable linear mixed-effects models in patients with Exfoliation Glaucoma.

Variables	CH	CRF	IOPcc	IOPg
	β (95% CI)	β (95% CI)	β (95% CI)	β (95% CI)
Age (per year)	−0.07 * (−0.13, −0.01)	−0.02 (−0.10, 0.05)	0.16 (−0.11, 0.42)	0.11 (−0.16, 0.38)
Male (vs. Female)	−0.35 (−1.21, 0.52)	0.51 (−0.58, 1.60)	2.54 (−1.30, 6.36)	2.61 (−1.31, 6.54)
G8	−0.09 (−0.30, 0.12)	0.20 (−0.06, 0.47)	0.89 (−0.05, 1.82)	0.93 (−0.03, 1.88)
CCI	−0.14 (−0.78, 0.50)	0.33 (−0.46, 1.13)	1.39 (−1.41, 4.20)	1.44 (−1.42, 4.31)
Mini-cog	0.02 (−0.20, 0.24)	−0.12 (−0.40, 0.16)	−0.37 (−1.34, 0.60)	−0.43 (−1.43, 0.56)
Medication score	−0.20 (−0.41, 0.01)	0.07 (−0.16, 0.30)	0.71 (−0.21, 1.63)	0.70 (−0.22, 1.62)

Values are regression coefficients (β) with 95% confidence intervals. All models were adjusted for age, sex, medication score, and glaucoma subtype. * *p* < 0.05. CI, confidence interval; CCI, Charlson Comorbidity Index; CH, corneal hysteresis; CRF, corneal resistance factor; IOPg, Goldmann-correlated intraocular pressure; IOPcc, corneal-compensated intraocular pressure.

**Table 5:** Association between geriatric assessment and ocular parameters using multivariable linear mixed-effects models with additional adjustment for mean deviation.

Variables	CH	CRF	IOPcc	IOPg
	β (95% CI)	β (95% CI)	β (95% CI)	β (95% CI)
Age (per year)	−0.04 *** (−0.05, −0.02)	−0.02 * (−0.05, −0.00)	0.05 (−0.01, 0.11)	0.02 (−0.05, 0.08)
Male (vs. Female)	−0.23 (−0.60, 0.14)	−0.14 (−0.60, 0.32)	0.41 (−0.95, 1.77)	0.19 (−1.21, 1.60)
G8	−0.01 (−0.11, 0.10)	0.18 ** (0.05, 0.31)	0.53 ** (0.14, 0.92)	0.61 ** (0.21, 1.02)
CCI	0.11 (−0.16, 0.38)	0.14 (−0.19, 0.48)	0.07 (−0.93, 1.07)	0.21 (−0.82, 1.25)
Mini-cog	0.01 (−0.09, 0.11)	−0.003 (−0.13, 0.12)	−0.06 (−0.42, 0.30)	−0.05 (−0.43, 0.33)
Medication score	−0.15 ** (−0.24, −0.06)	−0.02 (−0.12, 0.08)	0.30 (−0.07, 0.67)	0.20 (−0.18, 0.57)
Mean deviation, dB	0.03 ** (0.01, 0.05)	0.02 * (0.00, 0.04)	−0.03 (−0.11, 0.06)	0.001 (−0.08, 0.08)

Values are regression coefficients (β) with 95% confidence intervals. All models were adjusted for age, sex, medication score, and glaucoma subtype. * *p* < 0.05, ** *p* < 0.01, *** *p* < 0.001. CI, confidence interval; CCI, Charlson Comorbidity Index; CH, corneal hysteresis; CRF, corneal resistance factor; IOPg, Goldmann-correlated intraocular pressure; IOPcc, corneal-compensated intraocular pressure; MD, Mean deviation.

**Table 6:** Association between geriatric assessment and ocular parameters using multivariable linear mixed-effects models excluding age.

Variables	CH	CRF	IOPcc	IOPg
	β (95% CI)	β (95% CI)	β (95% CI)	β (95% CI)
Male (vs. Female)	−0.26 (−0.63, 0.11)	−0.21 (−0.66, 0.25)	0.32 (−1.00, 1.64)	0.06 (−1.31, 1.43)
G8	0.03 (−0.07, 0.14)	0.20 ** (0.07, 0.33)	0.44 * (0.07, 0.82)	0.56 ** (0.17, 0.94)
CCI	−0.00 (−0.27, 0.26)	0.03 (−0.30, 0.35)	0.13 (−0.82, 1.08)	0.14 (−0.84, 1.12)
Mini-cog	0.04 (−0.06, 0.14)	0.03 (−0.09, 0.15)	−0.05 (−0.41, 0.30)	−0.01 (−0.37, 0.36)
Medication score	−0.18 *** (−0.26, −0.09)	−0.02 (−0.11, 0.08)	0.39 * (0.05, 0.74)	0.29 (−0.06, 0.63)

Values are regression coefficients (β) with 95% confidence intervals. All models were adjusted for age, sex, medication score, and glaucoma subtype. * *p* < 0.05, ** *p* < 0.01, *** *p* < 0.001. CI, confidence interval; CCI, Charlson Comorbidity Index; CH, corneal hysteresis; CRF, corneal resistance factor; IOPg, Goldmann-correlated intraocular pressure; IOPcc, corneal-compensated intraocular pressure.

## Data Availability

The raw data supporting the conclusions of this article will be made available by the authors on request.

## Abbreviations

The following abbreviations are used in this manuscript:

IOP	Intraocular pressure
MCI	Mild cognitive impairment
GA	Geriatrics assessment
CCI	Charlson Comorbidity Index
CH	Corneal hysteresis
CRF	Corneal resistance factor
IOPg	Goldmann-correlated intraocular pressure
IOPcc	Corneal-compensated intraocular pressure
EXG	Exfoliation glaucoma
POAG	Primary open-angle glaucoma
ORA	Ocular Response Analyzer
MMSE	Mini-Mental State Examination
MoCA	Montreal Cognitive Assessment
